# Comparing cytotoxicity of propoxur and *Nepeta crispa* (Lamiales: Lamiaceae) essential oil against invertebrate (Sf9) and vertebrate (L929) cell lines

**DOI:** 10.14202/vetworld.2019.1698-1706

**Published:** 2019-11-04

**Authors:** Amirhossein Zahirnia, Mitra Boroomand, Hassan Nasirian, Aref Salehzadeh, Sara Soleimani-Asl

**Affiliations:** 1 Department of Medical Entomology, School of Medicine, Hamadan University of Medical Sciences, Hamadan, Iran; 2 Department of Medical Entomology and Vector Control, School of Public Health, Tehran University of Medical Sciences, Tehran, Iran; 3 Department of Anatomy, School of Medicine, Hamadan University of Medical Sciences, Hamadan, Iran

**Keywords:** biodegradable derivative, invertebrate cell line, plant essential oil, plant natural products, vertebrate cell line

## Abstract

**Background and Aim:**

Attempts to use the plant products are to be an appropriate option due to substantial concerns about human health and environmental problems of using synthetic pesticides. Therefore, the cytotoxicity of *Nepeta crispa* essential oil was compared with propoxur against invertebrate (Sf9) and vertebrate (L929) cell lines.

**Materials and Methods:**

The cell lines of Sf9 and L929 which were derived from the ovary glands of fall armyworm, *Spodoptera frugiperda* (Lepidoptera: Noctuidae) and mouse fibroblast cells, respectively, were obtained from the National Cell Bank of Pasteur Institute of Iran. About a number of 2 × 10^3^ cells were placed into the wells of 96-well plate experiments. Then, appropriate concentrations of essential oil of *N. crispa* plant and propoxur added to the wells. The cells were allowed to grow for 3-5 days and estimated the numbers of cells. The cells of control experiment wells contained only cells with dimethyl sulfoxide. All control and treatment experiments repeated at least four replicates.

**Results:**

Propoxur had negative effects on the viability of both invertebrate (Sf9) and vertebrate (L929) cell lines. The cytotoxicity of propoxur against invertebrate (Sf9) and vertebrate (L929) cell lines was gradually increased in accordance with propoxur concentrations. The cytotoxicity of *N. crispa* essential oil against vertebrate (L929) cell line was gradually decreased in accordance with plant concentrations, while the cytotoxicity of *N. crispa* essential oil against invertebrate (Sf9) cell line was strongly increased in accordance with plant concentrations.

**Conclusion:**

Plant essential oil not only had no negative effects but also had boosting effects on vertebrate cell viability. Essential oil of *N. crispa* plant had negative effects on invertebrate cell viability with the differences that the products derived from plants possessing of biodegradable and environmentally friendly derivatives, hydrolyzing rapidly in nature, and nearly having no destructive effects on environment, humans, or the mammals.

## Introduction

Attempts to use the plant products as natural pesticides usually based on plant essential oils are considered as a good candidate for the management of arthropod vectors of diseases and agricultural pests due to possessing of readily biodegradable and environmentally friendly derivatives [1-5]. There is a growing interest in the potential use of essential oils as a replacement for traditional herbicides and pesticides. Essential oils are complex natural products of plant origin exhibiting to have insecticidal, attractant, or repellent properties [6-8].

There are substantial concerns about human health and environmental problems of synthetic pesticides. Pesticides have potential side effects despite their importance to public health. Exposures to various types of insecticides may be a risk factor for cancers. Several undesirable effects of pesticides are maybe toxicity to non-target organisms, residues of pesticides, depletion of ozone layer, environmental pollution, and direct toxicity to users [2,9-12]. Insecticide resistance in agricultural pests and vectors of diseases is also maybe as a substantial problem in management programs of the pests and vectors [7,13-19].

One of the most natural aromatic plants in Iran is *Nepeta crispa* Willd. (Lamiales: Lamiaceae) that is popular in Iranian traditional medicine, especially with Hamadan Province people. *N. crispa* have insecticidal activity with antimicrobial and antifungal properties. The *N. crispa* plants are autochthonous of Iran climate, especially Hamadan Province [20-22].

Many studies have been conducted on the insecticidal properties of *N. crispa* plant, but a simultaneous comparative study about the cytotoxicity of essential oil of *N. crispa* with synthetic pesticides such as propoxur against the cell lines of invertebrates and vertebrates would be a particular of importance. Therefore, in this study, we compared the cytotoxicity of *N. crispa* essential oil with propoxur against invertebrates and vertebrates cell lines.

## Materials and Methods

### Ethical approval

The study was approved by the Ethics Committee of Hamadan University of Medical Sciences, Hamadan, Iran, with approval number: IR.UMSHA.REC.1396.320.

### Invertebrate (Sf9) and vertebrate (L929) cell line providing

Invertebrate (Sf9) and vertebrate (L929) cell lines were obtained from the National Cell Bank of Pasteur Institute of Iran. Cell line of Sf9 derived from the ovary glands of fall armyworm, *Spodoptera frugiperda* (Smith) (Lepidoptera: Noctuidae). Cell line of Sf9 is routinely cultured and maintained at 27°C in 5 ml of Grace’s insect cell culture medium in 25 cm^2^ culture flasks, enriched with 10% fetal bovine serum at Pasteur Institute of Iran. The doubling time of Sf9 cells was found to be 18-24 h under optimum conditions. Sf9 invertebrate cells were subcultured every 3 days. Cell line of L929 was derived from mouse fibroblast cells. It was maintained at 37°C in 3 ml of Dulbecco’s Modified Eagle Medium Media (Gibco^®^) in 25 cm^2^ culture flasks, enriched with 10% fetal bovine serum, and buffered with 4% sodium bicarbonate in an atmosphere of 5% carbon dioxide. The doubling time of L929 cells for cultures was approximately 24 h and the cell was subcultured every 6 day [23].

### Plant essential oil isolation

Technical grade of propoxur (white crystalline powder 97%) was purchased from Guangdong Company, China (Mainland). The aerial parts (foliage) of *N. crispa* (Lamiales: Lamiaceae) during their flowering stage were collected from Avicenna Medicinal Herbs Research Center, Hamadan Province of Iran in June 2017. The plant was confirmed by a voucher specimen (no. 72) in the Department of Pharmacognosy, School of Pharmacy, Hamadan University of Medical Sciences, Hamadan, Iran. A total of 1000 g powder of shade-dried aerial parts of *N. crispa* were subjected to hydrodistillation using a Clevenger-type apparatus for 4 h. The essential oil was dehydrated over anhydrous sodium sulfate and transferred into amber-colored vials to store in a refrigerator at 4°C for further work.

### Bioassay procedures

Essential oil (0.1 mg) of *N. crispa* plant was dissolved with 1 ml of dimethyl sulfoxide (DMSO) due to hydrophobic properties and then diluted with sterilized distilled water to prepare the concentrations of 10^−10^, 10^−9^, 10^−8^, 10^−7^, 10^−6^, 10^−5^, 10^−4^, and 10^−3^ ng/μL containing 0.00001, 0.0001, 0.001, 0.01, 0.1, 1, 10, and 100 ng/μL, respectively. Propoxur technical grade (97%) was dissolved with ethanol 96% to prepare the concentrations of 10^−10^, 10^−9^, 10^−8^, 10^−7^, 10^−6^, 10^−5^, 10^−4^, and 10^−3^ µg/μL containing 0.000097, 0.00097, 0.0097, 0.097, 0.97, 9.7, 97, and 970 µg/μL, respectively.

To consider the cytotoxicity of essential oil of *N. crispa* plant and propoxur against invertebrate (Sf9) and vertebrate (L929) cell lines, about 2 × 10^3^ cells/100 µl of culture medium were placed into the wells of a 96-well plate of treatment experiments and then appropriate concentrations of *N. crispa* essential oil and propoxur added to the wells. We allowed the cells to grow for 3-5 days and estimated the number of cells as described. Control cell wells contained only cells with 1µl/mL of DMSO. All treatment and control experiments repeated at least four replicates.

### Cell line number estimation

The base method for cell estimation is the Mossman method, which uses 3-(4,5-dimethylthiazol-2-yl)-2,5-difenyltetrazolium bromide (MTT, tetrazolium, compound). MTT is a quantitative coloring for living cells and cell proliferation, and it is a known method for *in vitro* cytotoxicity which measures the active metabolism of the cells. In this coloring solution, dehydrogenase enzyme reduced the MTT and produced blue formazan.

The wells of 96-well plates containing invertebrate (Sf9) and vertebrate (L929) cell lines were incubated with 10 µ MTT for 3 h at 36°C and 27°C, respectively. After the blue formazan and cells settled out and the supernatant was removed, 100 µ of DMSO was added to any well of 96-well plate, shaked for 15 min and then the absorbance of the solution read at 492 nm using ELISA reader [24].

### Statistical analysis

The cytotoxicity trend lines of *N. crispa* essential oil and propoxur against invertebrate (Sf9) and vertebrate (L929) cell lines were estimated by Microsoft Excel version 2013. The trend lines were drawn by clicking on graph line distribution and selecting “add trendline” option using procedure described in previous studies [17-28].The equations and R-squared values of the cytotoxicity trend lines were also calculated by Microsoft Excel. IBM SPSS statistics data editor version 24 was used for any statistical analyses. Wilcoxon signed-rank test was used for comparing cytotoxicity of essential oil of *N. crispa* plant and propoxur between control and treatments, and cross treatments of *N. crispa* plant and propoxur. p<0.05 was considered statistically significant.

## Results

### Cytotoxicity of *N. crispa* essential oil and propoxur

**Figure-1:**
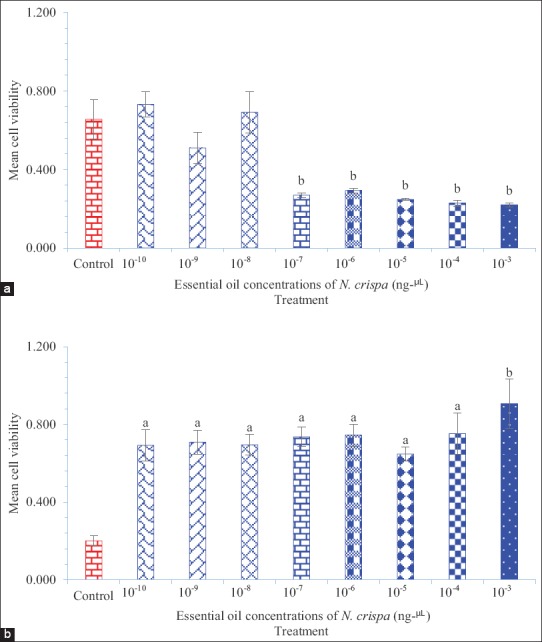
Cytotoxicity *Nepeta crispa* essential oil against invertebrate (Sf9) and vertebrate (L929) cell lines. (a) Essential oil of *N. crispa* plant against cell line of Sf9 and (b) essential oil of *N. crispa* plant against cell line of L929. Compared with control: (a) p<0.05 and (b) p<0.001. Cell line of Sf9 derived from the ovary gland of the fall armyworm, *Spodoptera frugiperda* (Smith) (Lepidoptera: Noctuidae). Cell line of L929 derived from mouse fibroblast cells.

**Figure-2:**
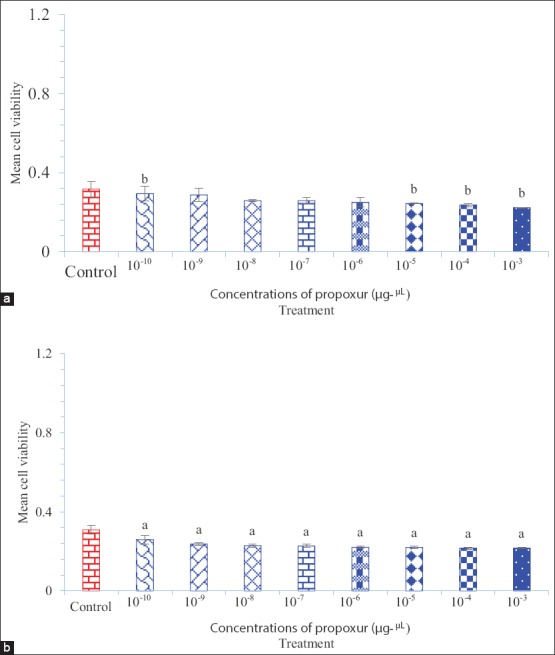
Cytotoxicity of propoxur against invertebrate (Sf9) and vertebrate (L929) cell lines. (a) Propoxur against cell line of Sf9 and (b) propoxur against cell line of L929. Compared with control: (a) p<0.05 and (b) p<0.001. Cell line of Sf9 derived from the ovary gland of the fall armyworm, *Spodoptera frugiperda* (Smith) (Lepidoptera: Noctuidae). Cell line of L929 derived from mouse fibroblast cells.

**Figure-3:**
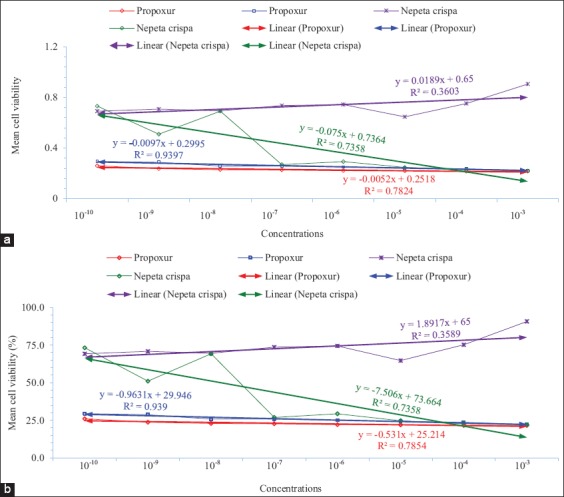
Cytotoxicity trend lines of *Nepeta crispa* essential oil (ng/μL) and propoxur (μg/μL) against invertebrate (Sf9) and vertebrate (L929) cell lines. (a) Normal data and (b) percentage. The trend lines were drawn by clicking on graph line distribution and selecting “add trendline” option. Cell line of Sf9 derived from the ovary gland of the fall armyworm, *Spodoptera frugiperda* (Smith) (Lepidoptera: Noctuidae). Cell line of L929 derived from mouse fibroblast cells.

Tables-1 and 2 show the data of *N. crispa* essential oil and propoxur cytotoxicity against invertebrate (Sf9) and vertebrate (L929) cell lines, respectively. Figures-1 and 2 show the cytotoxicity of *N. crispa* essential oil and propoxur against invertebrate (Sf9) and vertebrate (L929) cell lines, respectively. Figure-3 shows the cytotoxicity trend lines of *N. crispa* essential oil (ng/μL) and propoxur (μg/μL) against invertebrate (Sf9) and vertebrate (L929) cell lines. Table-3 shows the results of descriptive analysis and Wilcoxon signed-rank test between control and treatments, treatments and cross treatments of cytotoxicity of essential oil of *N. crispa* plant, and propoxur against invertebrate (Sf9) and vertebrate (L929) cell lines.

**Table-1:** Cytotoxicity of *Nepeta crispa* essential oil (ng/μL) against invertebrate (Sf9) and vertebrate (L929) cell lines.

Replicate		R_1_	R_2_	R_3_	R_4_	R_5_	R_6_	R_7_	R_8_
Control									
	Sf9	0.898	0.731	0.429	0.564	‒	‒	‒	‒
	L929	0.244	0.276	0.273	0.265	0.154	0.104	0.130	0.159
Treatment									
Concentration									
10^−10^	Sf9	0.769	0.561	0.865	0.732	‒	‒	‒	‒
	L929	0.594	0.556	0.578	0.278	0.858	0.862	0.935	0.873
10^−9^	Sf9	0.699	0.34	0.431	0.569	‒	‒	‒	‒
	L929	0.548	0.525	0.572	0.538	0.837	0.867	0.876	‒
10^−8^	Sf9	0.692	0.986	0.593	0.496	‒	‒	‒	‒
	L929	0.554	0.545	0.538	0.580	0.813	0.827	0.865	0.837
10^−7^	Sf9	0.239	0.292	0.268	0.277	‒	‒	‒	‒
	L929	0.560	0.588	0.575	0.75	0.83	0.859	0.872	0.854
10^−6^	Sf9	0.279	0.322	0.301	0.269	‒	‒	‒	‒
	L929	0.578	0.588	0.583	0.842	0.861	0.893	0.865	‒
10^−5^	Sf9	0.242	0.24	0.262	0.243	‒	‒	‒	‒
	L929	0.598	0.546	0.87	0.691	0.695	‒	‒	‒
10^−4^	Sf9	0.192	0.217	0.269	0.235	‒	‒	‒	‒
	L929	0.861	0.909	0.101	0.878	0.723	0.699	0.707	‒
10^−3^	Sf9	0.191	0.235	0.222	0.231	‒	‒	‒	‒
	L929	0.573	0.546	0.545	0.668	‒	‒	‒	‒

Cell line of Sf9 derived from the ovary gland of the fall armyworm, *Spodoptera frugiperda* (Smith) (Lepidoptera: Noctuidae). Cell line of L929 derived from mouse fibroblast cells. R=Replicate

**Table-2:** Cytotoxicity of propoxur (μg/μL) against invertebrate (Sf9) and vertebrate (L929) cell lines.

Replicate		R_1_	R_2_	R_3_	R_4_	R_5_	R_6_	R_7_	R_8_
Control									
	Sf9	0.429	0.256	0.274	0.307	‒	‒	‒	‒
	L929	0.429	0.256	0.274	0.307	0.377	0.319	0.287	0.226
Treatment									
Concentration									
10^−10^	Sf9	0.402	0.254	0.27	0.249	‒	‒	‒	‒
	L929	0.402	0.254	0.27	0.249	0.213	0.238	0.243	0.208
10^−9^	Sf9	0.263	0.232	0.264	0.390	‒	‒	‒	‒
	L929	0.263	0.242	0.244	0.253	0.21	0.22	0.228	0.236
10^−8^	Sf9	0.267	0.257	0.25	0.258	‒	‒	‒	‒
	L929	0.255	0.257	0.25	0.245	0.183	0.215	0.221	0.206
10^−7^	Sf9	0.244	0.272	0.263	0.235	‒	‒	‒	‒
	L929	0.244	0.247	0.243	0.194	0.215	0.219	‒	‒
10^−6^	Sf9	0.275	0.225	0.264	0.236	‒	‒	‒	‒
	L929	0.243	0.225	0.23	0.237	0.183	0.211	0.217	‒
10^−5^	Sf9	0.241	0.247	0.243	0.2437	‒	‒	‒	‒
	L929	0.238	0.229	0.225	0.223	0.2	0.199	0.226	‒
10^−4^	Sf9	0.226	0.225	0.261	0.231	‒	‒	‒	‒
	L929	0.226	0.225	0.221	0.229	0.187	0.209	0.216	‒
10^−3^	Sf9	0.226	0.219	0.221	0.222	‒	‒	‒	‒
	L929	0.212	0.225	0.219	0.211	0.215	‒	‒	‒

Cell line of Sf9 derived from the ovary gland of the fall armyworm, *Spodoptera frugiperda* (Smith) (Lepidoptera: Noctuidae). Cell line of L929 derived from mouse fibroblast cells. R=Replicate

**Table-3:** Results of Wilcoxon signed-rank test between cytotoxicity of *N. crispa* essential oil and propoxur against invertebrate (Sf9) and vertebrate (L929) cell lines.

Descriptive statistics									

Essential oil of *N. crispa* plant					Propoxur				
	
Concentration	Mean	Standard deviation	Mean	Standard deviation	Concentration	Mean	Standard deviation	Mean	Standard deviation
			
	Sf9		L929			Sf9		L929	
Control	0.6555	0.2035	0.2006	0.0709	Control	0.3165	0.0779	0.3094	0.0660
Treatment					Treatment				
10^−10^	0.7318	0.1269	0.6918	0.2270	10^−10^	0.2938	0.0727	0.2596	0.0611
10^−9^	0.5098	0.1574	0.7080	0.1748	10^−9^	0.2873	0.0701	0.2370	0.0173
10^−8^	0.6918	0.2119	0.6949	0.1515	10^−8^	0.2580	0.0085	0.2290	0.0269
10^−7^	0.2690	0.0223	0.7360	0.1391	10^−7^	0.2580	0.0198	0.2270	0.0212
10^−6^	0.2928	0.0236	0.7443	0.1516	10^−6^	0.2500	0.0354	0.2209	0.0200
10^−5^	0.2468	0.0102	0.6471	0.1039	10^−5^	0.2437	0.0031	0.2200	0.0148
10^−4^	0.2283	0.0324	0.7522	0.3007	10^−4^	0.2358	0.0170	0.2161	0.0145
10^−3^	0.2198	0.0199	0.9065	0.3632	10^−3^	0.2220	0.0036	0.2164	0.0057

**Wilcoxon signed-rank test**									

**Analysis between treatments and control**									

**Essential oil of *N. crispa* plant**						**Propoxur**			
	
		**Mean ranks**		**Z**	**p-value (two tailed)**	**Mean ranks**		**Z**	**p-value (two tailed)**
	
		**Negative**	**Positive**			**Negative**	**Positive**		

Sf9									
	10^−10^	2.0	3.0	−0.365^b^	0.715	2.5	0.001	−1.826^c^	0.068
	10^−9^	3.5	1.5	−0.730^c^	0.465	2.3	3.0	−0.730^c^	0.465
	10^−8^	2.0	3.0	−0.365^c^	0.715	3.0	1.0	−1.461^c^	0.144
	10^−7^	2.5	0.001	−1.826^c^	0.068	3.0	1.0	−1.461^c^	0.144
	10^−6^	2.5	0.001	−1.826^c^	0.068	3.0	1.0	−1.461^c^	0.144
	10^−5^	2.5	0.001	−1.826^c^	0.068	2.5	0.001	−1.826^c^	0.068
	10^−4^	2.5	0.001	−1.826^c^	0.068	2.5	0.001	−1.826^c^	0.068
	10^−3^	2.5	0.001	−1.826^c^	0.068	2.5	0.001	−1.826^c^	0.068
L929									
	10^−10^	0.001	4.5	−2.521^b^	0.012	4.5	0.001	−2.521^c^	0.012
	10^−9^	0.001	4.0	−2.366^b^	0.018	5.0	1.0	−2.380^c^	0.017
	10^−8^	0.001	4.5	−2.521^b^	0.012	5.0	1.0	−2.380^c^	0.017
	10^−7^	0.001	4.5	−2.521^b^	0.012	3.5	0.001	−2.201^c^	0.028
	10^−6^	0.001	4.0	−2.366^b^	0.018	4.0	0.001	−2.371^c^	0.018
	10^−5^	0.001	4.5	−2.521^b^	0.012	4.0	0.001	−2.366^c^	0.018
	10^−4^	1.0	4.5	−2.197^b^	0.028	4.0	0.001	−2.366^c^	0.018
	10^−3^	0.001	2.5	−1.826^b^	0.068	3.0	0.001	−2.023^c^	0.043
		Sf9				L929			
Analysis between the treatments of essential oil of *N. crispa* plant and propoxur									
10^−10^	10^−10^	0.001	2.5	−1.826^b^	0.068	0.001	0.001	0.0001^a^	1.000
10^−9^	10^−9^	3.0	2.0	−0.365^c^	0.715	1.0	2.5	−1.069^b^	0.285
10^−8^	10^−8^	2.0	2.7	−1.095^b^	0.273	0.001	1.5	−1.342^b^	0.180
10^−7^	10^−7^	2.5	0.001	−1.826^c^	0.068	0.001	2.0	−1.604^b^	0.109
10^−6^	10^−6^	2.5	0.001	−1.826^c^	0.068	1.0	2.5	−1.069^b^	0.285
10^−5^	10^−5^	2.5	0.001	−1.826^c^	0.068	0.001	2.5	−1.841^b^	0.066
10^−4^	10^−4^	3.0	1.0	−1.461^c^	0.144	0.001	1.5	−1.342^b^	0.180
10^−3^	10^−3^	2.5	0.001	−1.826^c^	0.068	2.0	2.7	−1.095^b^	0.273
Cross analysis between the treatments of essential oil of *N. crispa* plant and propoxur									
10^−10^	10^−10^	0.00	2.50	−1.826^b^	0.068	0.001	4.50	−2.521^b^	0.012
10^−9^	10^−9^	0.00	2.50	−1.826^b^	0.068	0.001	4.00	−2.371^b^	0.018
10^−8^	10^−8^	0.00	2.50	−1.826^b^	0.068	0.001	4.50	−2.524^b^	0.012
10^−7^	10^−7^	1.50	2.83	−1.289^b^	0.197	0.001	3.50	−2.201^b^	0.028
10^−6^	10^−6^	0.00	2.50	−1.826^b^	0.068	0.001	4.00	−2.366^b^	0.018
10^−5^	10^−5^	2.00	3.00	−0.365^b^	0.715	0.001	4.00	−2.371^b^	0.018
10^−4^	10^−4^	3.25	1.75	−0.552^c^	0.581	1.00	4.50	−2.197^b^	0.028
10^−3^	10^−3^	4.00	2.00	−0.365^b^	0.715	0.001	2.50	−1.826^b^	0.068

a The sum of negative ranks equals the sum of positive ranks,

b based on negative ranks and

c based on positive ranks. The p-value of significant (p<0.05) is shown in bold font style. Cell line of Sf9 derived from the ovary gland of the fall armyworm, *Spodoptera frugiperda* (Smith) (Lepidoptera: Noctuidae). Cell line of L929 derived from mouse fibroblast cells. R=Replicate. *N. crispa*: *Nepeta crispa*

The cytotoxicity of essential oil of *N. crispa* plant against invertebrate (Sf9) cell line was strongly increased with an intensive increasing slope in accordance with essential oil concentrations of *N. crispa* plant from 10^−10^ to 10^−3^ µg/μL (Figures-1a and 3), while the cytotoxicity of essential oil of *N. crispa* plant against vertebrate (L929) cell line was gradually decreased with a moderately increasing slope in accordance with essential oil concentrations of *N. crispa* plant from 10^−10^ to 10^−3^ µg/μL (Figures-1b and 3). Wilcoxon signed-rank test revealed significant differences between the treatments of 10^−10^ and 10^−4^ essential oil concentrations of *N. crispa* plant against vertebrate (L929) cell line with control (p<0.05) (Table-3 and Figure-1b). Wilcoxon signed-rank test did not show a significant difference between the treatment of 10^−3^ essential oil concentration of *N. crispa* plant against vertebrate (L929) cell line with control (p=0.068) (Table-3), even though there was a significant difference at p<0.001 level between the treatments of 10^−3^ essential oil concentration of *N. crispa* plant against vertebrate (L929) cell line with control (Figure-1b). Wilcoxon signed-rank test did not show significant differences between the treatments of 10^−10^ and 10^−3^ essential oil concentrations of *N. crispa* plant against invertebrate (Sf9) cell line with control (p>0.05) (Table-2 and Figure-1a), even though there were significant differences at p<0.001 level between the treatments of 10^−7^ and 10^−3^ essential oil concentrations of *N. crispa* plant against cell line of Sf9 (Figure-1a).

The cytotoxicity of propoxur against invertebrate (Sf9) and vertebrate (L929) cell lines was gradually increased with a relatively low decreasing slope in accordance with propoxur concentrations from 10^−10^ to 10^−3^ µg/μL (Figures-2a, b and 3). Wilcoxon signed-rank test did not show significant differences between the treatments of 10^−10^ and 10^−3^ concentrations of propoxur against invertebrate (Sf9) cell line with control (p>0.05) (Table-3 and Figure-2a), even though there was a significant difference at p<0.001 level between the treatments of 10^−10^ and 10^−5^ to 10^−3^ concentrations of propoxur against invertebrate (Sf9) cell line (Figure-2a). There were also significant differences between the treatments of 10^−10^ and 10^−3^ concentrations of propoxur against vertebrate (L929) cell line with control (p<0.05) (Table-3 and Figure-2b).

### Comparing of *N. crispa* essential oil and propoxur cytotoxicity

The cytotoxicity of essential oil of *N. crispa* plant against invertebrate (Sf9) cell line was strongly increased with an intensive increasing slope in accordance with essential oil concentrations of *N. crispa* plant, while the cytotoxicity of essential oil of *N. crispa* plant against vertebrate (L929) cell line was gradually decreased with a moderately increasing slope in accordance with essential oil concentrations of *N. crispa* plant. The cytotoxicity of propoxur against invertebrate (Sf9) and vertebrate (L929) cell lines was gradually increased with a relatively low decreasing slope in accordance with propoxur concentrations (Figure-3).

Wilcoxon signed-rank test did not show significant differences between separatel*y* treatments and cros*s* treatments of *N. crispa* plant and propoxur concentrations against invertebrate (Sf9) and vertebrate (L929) cell lines, and invertebrate (Sf9) cell line, respectively (p>0.05), even though there were significant differences between cros*s* treatments (10^−10^–10^−4^) of *N. crispa* plant and propoxur concentrations against vertebrate (L929) cell line (p<0.05) (Table-3).

## Discussion

Based on the results of the study, propoxur had negative effects on the viability of both invertebrate (Sf9) and vertebrate (L929) cell lines. These results confirm the reports of the previous studies that concluded insecticides decreased growth of the invertebrate and vertebrate cell lines [29,30]. Compared with the propoxur treatment experiments, the highest rate of cell viability was observed in the control group which did not receive any toxic agent.The viability of cell lines which were exposed to the different concentrations of propoxur was lower than control group and decreased with increasing concentrations of propoxur. The negative effects of propoxur on the viability of both invertebrate (Sf9) and vertebrate (L929) cell lines were also confirmed by Wilcoxon signed-rank test by observing significant differences between the treatments of 10^−5^-10^−3^ and 10^−8^-10^−3^ concentrations of propoxur with control group against the invertebrate (Sf9) and vertebrate (L929) cell lines, respectively, at p<0.05 or p<0.001 levels (Table-3).

Unlike propoxur, essential oil of *N. crispa* plant did not have negative effects on the viability of vertebrate (L929) cell line. Compared with the control group, the highest rate of cell viability was observed in the experiment treatments which were treated with essential oil of *N. crispa* plant. The viability of vertebrate (L929) cell line which was exposed to different essential oil concentrations of *N. crispa* plant was higher than control group and increased with increasing essential oil concentrations of *N. crispa* plant. The boosting effects of experiments of *N. crispa* plant on the viability of vertebrate (L929) cell line were also confirmed by Wilcoxon signed-rank test by observing significant differences between the treatments of 10^−10^ and 10^−3^ essential oil concentrations of *N. crispa* plant with propoxur treatments of 10^−10^-10^−3^ concentrations and control group against the vertebrate (L929) cell line at p<0.05 or p<0.001 levels. In addition to some previous benefits of the natural products deriving from natural plants [31,32], this boosting effects of essential oil of *N. crispa* plant on the viability of cell line of vertebrates may be considered as the new benefits of the products deriving from natural plants.

Like propoxur, essential oil of *N. crispa* plant had negative effects on the viability of invertebrate (Sf9) cell lines with the differences that the products deriving from natural plants possessing of readily biodegradable and environmentally friendly derivatives, hydrolyzing rapidly in nature, and nearly having no destructive effects on environment, humans, or the mammals [1,2]. In addition, the application of essential oil concentrations of *N. crispa* plant (ng/μL) was 1000 folds lower than concentrations of propoxur (μg/μL). The viability of invertebrate (Sf9) cell lines which were exposed to essential oil concentrations of 10^−9^ and 10^−7^-10^−3^ *N. crispa* plant was lower than control group and decreased with increasing essential oil concentrations of *N. crispa* plant.

The negative effects of essential oil of *N. crispa* plant on the viability of invertebrate (Sf9) cell lines were also confirmed by Wilcoxon signed-rank test by observing a significant difference between the treatments of 10^−7^ and 10^−3^ essential oil concentrations of *N. crispa* plant with control group against the invertebrate (Sf9) cell lines at p<0.001 level. In addition to that, the food chain and ecosystem contamination with pesticides, non-target organism extinction, insecticide resistance of insects, mutation, and pollution of water and soil have been critical problems using pesticides in the past decades. However, with the application of the products deriving from natural plants, there is no concern about their potential side effects such as toxicity to non-target organisms, residues of pesticides, depletion of ozone layer, environmental pollution, and direct toxicity to users. We will not encounter probably some psychotic disorders, neurological diseases and fatigue of muscles or cancers, and face up to severe insecticide effects on performance of vertebrate reproductive system [2,9-12]. Some studies did not encounter a substantial problem of insecticide resistance in arthropods of the vectors of diseases and agricultural pests [7,13-19].

## Conclusion

Based on the results of the study, propoxur had negative effects on the viability of both invertebrate and vertebrate cell lines. Unlike propoxur, essential oil of *N. crispa* plant did not have negative effects on the viability of vertebrate cell line. The viability of vertebrate cell line was increased with increasing essential oil concentrations of *N. crispa* plant. This boosting effect of *N. crispa* plant on the viability of vertebrate cell line may be considered as the new benefits of the products deriving from natural plants.

## Authors’ Contributions

AS and AZ planned the project. MB and HN conducted the experimental laboratory work and analyzed the data. SS assisted in analyzing of experimental data. AS and HN wrote the manuscript. AS supervised the project. All authors discussed the results and implications and commented on the manuscript at all stages. All authors read and approved the final manuscript.
